# Pancreatic cancer risk in hereditary pancreatitis

**DOI:** 10.3389/fphys.2014.00070

**Published:** 2014-02-20

**Authors:** Frank U. Weiss

**Affiliations:** Department of Internal Medicine A, University Medicine GreifswaldGreifswald, Germany

**Keywords:** cancer risk, pancreatitis, hereditary pancreatitis, PRSS1, chronic inflammation

## Abstract

Inflammation is part of the body's immune response in order to remove harmful stimuli—like pathogens, irritants or damaged cells—and start the healing process. Recurrent or chronic inflammation on the other side seems a predisposing factor for carcinogenesis and has been found associated with cancer development. In chronic pancreatitis mutations of the cationic trypsinogen (*PRSS1*) gene have been identified as risk factors of the disease. Hereditary pancreatitis (HP) is a rare cause of chronic pancreatic inflammation with an early onset, mostly during childhood. HP often starts with recurrent episodes of acute pancreatitis and the clinical phenotype is not very much different from other etiologies of the disease. The long-lasting inflammation however generates a tumor promoting environment and represents a major risk factor for tumor development This review will reflect our knowledge concerning the specific risk of HP patients to develop pancreatic cancer.

## Introduction

Chronic pancreatitis (CP) and carcinoma of the pancreas are common in Western countries. Incidence rates of CP range from 2 to 23 per 100,000 and are around 10 per 100,000 for the incidence and death rate of pancreatic cancer (Dufour and Adamson, 2003; Lévy et al., 2006; Ferlay et al., 2010; Spanier et al., 2013). However, not all CP develops into cancer, even not in the very long-standing cases, and the majority of patients presenting with pancreatic carcinoma have no history of CP.

In a recent pooled analysis by the International Pancreatic Cancer Case-Control Consortium (PanC4) Duell et al. reviewed a total of 5048 cancer cases and 10947 controls. Interestingly, only 6.2% of pancreatic cancer patients reported a history of pancreatitis. Duell et al. calculated a ~5.6-fold increased pancreatic cancer risk in patients with a history of pancreatitis (Duell et al., 2012). In the first two years following diagnosis of pancreatitis, the risk is even higher (OR: 13.6), probably reflecting increased likelihood of cancer diagnosis in people undergoing medical investigations, and possible misdiagnosis of pancreatic cancer as pancreatitis. The type of pancreatitis was not determined in most of the evaluated studies, preventing a more detailed analysis of the specific risk of acute vs. CP.

Much more than a single inflammatory event, the recurrent or persistent chronic inflammation is regarded as an important risk factor for cancer development, not only in the pancreas, but in many different organs (Mantovani et al., 2008). Observations that tumors often arise at sites of chronic inflammation were first made in the nineteenth century (Balkwill and Mantovani, 2001). Since that time several lines of evidence, based on histologic findings of inflammatory cells in tumor samples and also genetic and molecular analyses have supported the general concept that inflammation and cancer are linked. In addition, epidemiologic studies have shown that chronic inflammation is associated with the development of several types of cancer. Factors that drive the chronic inflammation process are many-fold and include toxins like cigarette smoke, alcohol, microbial infection (helicobacter pylori), autoimmune diseases (M. Chron), inflammatory conditions of unknown origin, a genetic predisposition (hereditary pancreatitis) or a combination of several factors.

Numerous studies which analyzed the pancreatic cancer risk of CP patients reported considerably different results, probably reflecting methodological variation concerning the recruitment, diagnosis and evaluation of patients. This review will mainly focus on the question if pancreatic cancer is especially frequent in those patients that are predisposed to CP by the presence of a *PRSS1* mutation.

## Hereditary pancreatitis

Hereditary pancreatitis is a rare cause of CP with an estimated frequency of 0.3 /100.000 in Western countries. In 1952, Comfort and Steinberg reported a family with hereditary CP over three generations. Affected patients had chronic relapsing pancreatitis with an unusual early onset of the disease (5–23 years) (Comfort and Steinberg, 1952). In 1996 Whitcomb et al. identified from a large HP family with an autosomal dominant inheritance pattern a first genetic defect of the cationic trysinogen gene (*PRSS1*) through sequencing analysis of the 7q35 chromosome region. They identified a G to A transition in exon 3 of the PRSS1 gene that encodes the replacement of Arginine 122 by Histidine (Whitcomb et al., 1996). Trypsins are digestive enzymes that are synthesized and secreted in large amounts by the acinar cells of the exocrine pancreas. Three different trypsinogen isoforms are known and cationic trypsinogen represents 2/3 of the total amount of trypsinogen in the pancreatic juice. Anionic trypsinogen accounts for another 1/3 of the trypsinogen, whereas mesotrypsin is expressed only in small traces. Trypsinogens are synthesized as enzymatically inactive pro-enzymes or zymogens that are stored and released from the secretory compartment of the acinar cell. Under physiological conditions trypsinogens are activated in the duodenum by enterokinase, which is produced by cells of the duodenal mucosa and which cleaves the N-terminal activation peptide bond and releases the enzymatic activity of trypsins. Trypsin is the main digestive enzyme of the gastrointestinal tract and has autoactivation as well as autolysis properties. Influenced by ambient pH and calcium concentration the protein may therefore either tend to self-activation or self-destruction. Subtle changes in the trypsin protein structure seem sufficient to disrupt the mechanism of normal trypsin activation leading to increased premature intrapancreatic trypsin activation or impaired inactivation. Both ways *PRSS1* mutations may lead to enhanced trypsin activity which eventually increases the risk for recurrent pancreatic injury and inflammation. Since 1996 more than 30 different PRSS1 mutations have been identified (www.uni-leipzig.de/pancreasmutation). The majority of these mutations were reported only in one or a few families and the biochemical analysis of these mutations gave valuable insights in the disease mechanism. Some mutations like K23R, D22G, or D19A are localized in the area where enterokinase activation of trypsinogen occurs. These mutations were found to facilitate trypsin autoactivation (Geisz et al., 2013).

Autoactivation of cationic trypsinogen is also influenced by chymotrypsin C (CTRC), which opposes the trypsin activity by promoting trypsinogen and trypsin degradation (Szmola and Sahin-Tóth, 2007). Chymotrypsin C selectively cleaves the Leu81-Glu82 peptide bond within the Ca2+ binding loop of cationic trypsin. Further degradation and inactivation is then achieved through tryptic cleavage of the Arg122-Val123 peptide bond. Therefore, mutation of either Leu81 or Arg122 blocks chymotrypsin C-mediated trypsin degradation (Szabó and Sahin-Tóth, 2012). The mechanistic basis of increased trypsinogen activation involves either resistance to degradation (N29I, N29T, V39A, R122C, and R122H) and/or increased N-terminal processing by CTRC (A16V and N29I). In hereditary pancreatitis the CTRC-dependent control of cationic trypsinogen autoactivation is disturbed giving rise to intrapancreatic trypsinogen activation. Most frequent *PRSS1* mutations R122H and N29I lead with high penetrance (~80%) to CP, in most cases with an early onset of symptoms. The A16V and R122C mutants were found to have a more variable disease penetrance ~40–50% (De Las Heras-Castaño et al., 2009; Grocock et al., 2010). Apart from some variation in disease penetrance the clinical phenotypes of these most relevant HP mutations seem rather comparable and—with the exception of an early onset—resemble the same features of CP of other etiologies.

Lowenfels and colleagues from the International Hereditary Pancreatitis Study Group were one of the first to review the medical records of 246 patients with a diagnosis of HP. Comparison of observed and expected frequency of cancer in this historical group of patients revealed and standardized incidence ratio (SIR) of pancreatic cancer of 53 (95%CI: 23–105). In those individuals that developed pancreatic cancer the mean age at onset of symptoms of pancreatitis was 17.3 ± 6.9 years and mean age at diagnosis of pancreatic cancer was 56.9 ±11.2 years, indicating a high risk of pancreatic cancer several decades (39.6 ± 9.7 years) after the initial onset of pancreatitis(Lowenfels et al., 1997). The risk was not different in males or in females or for different nationalities and the cumulative risk in these patients until the age of 70 was 40%. The diagnosis of HP in the study was mainly based on early onset of pancreatitis, a positive family history and the absence of other known causes of pancreatitis. Today we know that many HP patients have an underlying causative *PRSS1* mutation, but at the time of the study by Lowenfels the genetic testing for *PRSS1* had only just started and therefore could not yet be systematically analyzed.

Such a genotype-phenotype correlation was done in 2004 by Howes et al. on behalf of the European registry of hereditary pancreatitis and pancreatic cancer (EUROPAC) (Howes et al., 2004). Their study cohort comprised 112 families (418 individuals) from 14 countries and included 52% R122H-families, 21% N29I-families, 4% A16V-families and 19% without detectable mutation. The high mutation rate of 81% in HP was much higher than previously reported and presumably due to the strict diagnostic criteria of HP by the EUROPAC group. The authors confirmed that onset of symptoms starts at young age for R122H mutation carriers with a median onset at 10 (95%CI: 8–12) and 14.5 (95%CI: 10–21) for mutation negative patients. Interestingly time to development of exocrine and endocrine failure showed no significant differences, neither by mutation status nor by gender. Still the cumulative risk for exocrine failure or diabetes is much higher in HP (60.2 and 68.6%) than in idiopathic or alcoholic pancreatitis patients. Pancreatic cancer was diagnosed in 26 (6%) patients and arose in individuals carrying any of the common mutations as well as in *PRSS1*-mutation negative families (14x R122H, 7x N29I, 1x A16V and 4x no *PRSS1* mutation). The time to develop cancer was not influenced by mutation status, gender or if the mutation was transmitted from the father or the mother. The study further showed that the cumulative risk of pancreatic cancer is rather negligible until the age of 50 (3.4%) in both sexes. However, after 50 years the risk of pancreatic cancer rises considerably, reaching 18.8% at 70 years and 33.3% at 80 years. In other words: the cumulative risk of pancreatic cancer after onset of symptoms slowly increases from 1.5% at 20 years and 2.5% at 30 years after first symptoms to 25.3% at 60 years and 44% at 70 years after the onset of the disease. The calculated SIR of pancreatic cancer in the EUROPAC cohort after correction for age, smokers, nationality and surgical intervention, was 67 (95%CI: 50–82).

In a national series in 2008 Rebours et al. investigated the SIR of pancreatic adenocarcinoma in an exhaustive cohort of French HP patients (Rebours et al., 2012). In their nation-wide survey genetic laboratories, pediatricians and gastroenterologists contributed 200 individuals from 78 families with know *PRSS1* mutation or the diagnosis of recurrent acute or CP in the absence of known precipitating factors. *PRSS1* mutations were present in 68% (78% R122H, 12% N29I, 10% others) of the study cohort and again the *PRSS1* mutation type did not correlate with the development of pancreatic cancer, which was diagnosed in ten individuals at a median age of 55. The cumulative risk at age 50 was ~10% and increased to ~50% at age 75. The SIR of pancreatic cancer in the French cohort was 87 (95%CI: 42–113) for the whole population and seemed higher in females (142; 95%CI: 38–225) compared to males (69; 95%CI: 25–150). Whereas Lowenfels et al. also found a slightly higher SIR in females (73 vs. 46) the results from the EUROPAC study indicated a higher SIR in men (72 vs. 60). A gender impact therefore seems not generally operative in the development of pancreatic adenocarcinoma.

In comparison to the general population HP patients clearly carry an increased absolute risk of developing pancreatic cancer. Smoking was identified as a main associated risk factor and HP patients therefore should be strongly advised to stay away from tobacco consumption. Diabetes and calcifications are also more frequently seen in patients that develop pancreatic cancer, probably indicating a correlation of the cancer risk not only with the duration but also with the severity of pancreatitis.

## Pancreatic cancer risk in sporadic pancreatitis of mutation carriers

In the clinical situation HP is diagnosed mainly in patients with idiopathic recurrent acute or CP families. However, sometimes also sporadic cases without a corresponding family history have a positive finding of an HP mutation. Inheritance from unaffected carrier parents, uncertain paternity and spontaneous *de novo* mutations must be considered in such cases (Simon et al., 2002). A recent study by Hamoir et al. identified a total of 17.4% carriers of *CFTR*, *PRSS1*, or *SPINK1* mutations in a cohort of 351 Belgium patients with idiopathic recurrent or CP and no family history (Hamoir et al., 2013). The authors claim that the clinical features were not influenced by the presence of a gene mutation except for an earlier age at onset and a higher incidence of pancreatic cancer, especially in patients with a *CFTR* mutation (four cancer patients had *CFTR* mutations, one a *PRSS1* mutation). The SIR for pancreatic cancer in their cohort was 26.5 (95%CI: 8.6–61.9). However, all cancer patients were also smokers. The authors suggest to “include patients with *CFTR* variants presenting with risk factors in a screening and surveillance program and to strongly advise them not to smoke.” Three of the four cancer patients with CFTR mutation carried the p.L997F mutation (2× compound heterozygous, 1× heterozygous) which also had been identified at high frequency in patients with recurrent idiopathic pancreatitis (Gomez Lira et al., 2000). Whereas there is no disagreement concerning the adequacy of a non-smoking advice other reports find a modest increased risk for carriers of disease-relevant *CFTR* mutations (OR:1.4; 95%CI: 1.04–1.89) and are more reluctant concerning the role of *CFTR* mutations as risk factors of pancreatic cancer (Whitcomb, 2004; McWilliams et al., 2010).

## Chronic inflammation and cancerogenesis

The link between CP and pancreatic cancer is unknown to date, but several signaling pathways were identified to become activated in the inflamed pancreas which may trigger cellular transformation and ultimately stimulate the development of pancreatic cancer.

It is generally accepted that inflammation results in repeated DNA damage, error-prone repair-mechanisms and the progressive accumulation of genetic mutations. In the pancreas pre-cancerous histologic changes have been described that are associated with a sequential accumulation of genetic defects. These pancreatic intra-epithelial neoplasms (PanIN) are present in sporadic pancreatic adenocarcinomas and also in patients with a history of CP. Histologically, PanINs progress through stages of increasing architectural and cytological atypia, starting from a low grade dysplasia (PanIN-1A, PanIN-1B) to moderate dysplasia (PanIN-2) and to high grade dysplasia (PanIN-3). First genetic mutations seen in the early stages include *kRas* mutations, followed by *p16/CDKN2A*, *TP53*, and *SMAD4/DPC4* (Hruban et al., 2004). Mutations in all four genes have been recognized as driver mutations that trigger neoplastic transformation and tumor progression (Korc, 2010). In a mouse model kRas mutations were shown to give rise to pancreatic intraepithelial neoplasms and pancreatic cancer and that concomitant mutation of p16, p53 or smad4 greatly enhanced the process of carcinoma formation (Hingorani et al., 2005). These mutations are more frequent in advanced PanIN stages and are well-characterized in invasive pancreatic carcinoma.

Signaling mechanisms involving Hedgehog and Notch, as well as cyclooxygenase 2 (*COX-2*) have also been implicated in the triggering mechanisms that stimulate the generation of pancreatic cancer from pancreatic inflammation (Maitra et al., 2002; Avila and Kissil, 2013; Hamada et al., 2013). COX-2 mediates prostaglandine synthesis which triggers cell proliferation and cytokine synthesis. Cox-2 inhibitors have been demonstrated to have anti-cancer effects and are effective especially in patients with cancers that have a high COX-2 expression. Extensive inflammation exposes the organ tissue to pro-inflammatory cytokines and reactive oxygen species. Local production of both may activate cellular protective mechanisms, including apoptosis and regenerating mechanisms that stimulate cell proliferation in order to rebuild the lost tissue structure. Increased proliferation in the presence of elevated concentrations of potential mutagens such as reactive oxygen species may set an environment where growth promoting mutations accumulate and provide selective growth advantage for individual cell clones.

Another signaling pathway that has been suggested to drive cancerogenesis from inflammation involves NFkB (Karin, 2006). Important cancer-associated genes, such as c-myc, jun B Cyclin D1, TP53, and VEGF are under the control of this transcription factor. In addition it's a major factor controlling the ability of malignant cells to resist apoptosis-based tumor-surveillance mechanisms.

## Perspective

### Pancreatic cancer surveillance

Today there is no rationale for early diagnostic screening of pancreatic cancer in the general population. It's a rare disease, specific diagnostic markers are missing and a survival benefit of such screening programs has nowhere been demonstrated. However, pancreatic cancer screening is recommended for families and individuals at an elevated risk. Counseling and surveillance guidelines have been established that recommend screening studies as part of peer-reviewed protocols with scientific evaluation and human subjects protection(Brand et al., 2007). Candidates for pancreatic cancer surveillance should carry a >10-fold increased risk for developing pancreatic cancer, which includes individuals with HP.

### Surgery

Generally, the survival rate for patients with CP is poor (Jupp et al., 2010). CP patients tend to die of other causes such as smoking related cancers, cardiovascular disease and alcoholic liver cirrhosis. The potential cancer risk of a persistent inflammation may suggest beneficial effects of anti-inflammatory therapy or surgery for CP. A recent retrospective multicenter study from Japan investigated associated factors with the pancreatic cancer risk in 506 patients with CP (Ueda et al., 2013). Nineteen of 506 enrolled patients developed pancreatic cancer (3.7%) with a SIR of 11.8 (95% CI, 7.1–18.4). Interestingly, among 9 patients with HP, no patient developed pancreatic cancer (follow-up period: 3.4–43.8 years, median, 8.4 years). Among the 352 CP patients who had not received surgical treatment a total of 18 patients (5.1%) developed pancreatic cancer, which otherwise occurred in only 1 (0.7%) of the 147 patients who had undergone surgery for CP. Apparently surgery for CP inhibits the development of pancreatic cancer in those patients.

In addition the study confirmed that patients who continued to drink alcohol after the diagnosis of CP showed a significant higher incidence of pancreatic cancer than those who stopped drinking.

### Biomarker

The goal for diagnostic screening is the identification of early cancer lesions before the onset of metastasis and tissue invasion. Until today no biomarker in plasma or serum has generally been recommended for screening or diagnosing of pancreatic cancer and there is an urgent need to identify novel markers of pancreatic cancer. The search is on for new strategies that help to improve the sensitivity and specificity of diagnostic procedures.

One example is a study of Yokoi et al. who analyzed proteins from circulating mononuclear cells (MNC) to identify surrogate markers of pancreatic cancer (Yokoi et al., 2011). Continuous interactions between tumor cells and host stroma cells is a fundamental requirement for tumor cell growth, invasion, and metastasis (Fidler et al., 2007). In histologic stainings the stroma typically occupies 70–90% of pancreatic tumors. Among the cellular components of the stroma, MNCs are believed to play a central role in the progression and chemoresistance of tumors (Condeelis and Pollard, 2006; Noonan et al., 2008). Circulating MNCs, such as monocytes and/or macrophages, are recruited into the tumor microenvironment, where they extravasate and differentiate into tumor-associated macrophages (TAMs) (Shojaei et al., 2008). Even small tumors could generate a detectable immune response that may include changes in protein content or phosphorylation of MNCs. Analysing circulating MNCs in a nude mouse model of orthotopic human pancreatic cancer, Yokoi et al. found significant higher Src-expression (c-src tyrosinkinase) and phosphorylation in MNCs from mice bearing tumors. The identified surrogate marker Src may not be a convincing finding so far, but circulating MNCs may represent a good source for the identification of novel biomarkers of early tumor development.

In summary HP markedly increases the risk for pancreatic adenocarcinoma. *PRSS1* and other pancreatitis-associated gene mutations are not directly important in the development of pancreatic cancer, but rather lead to a high-risk inflammatory milieu for the accumulation of oncogenic mutations. The risk is potentiated by known cofactors such as tobacco smoking and, likely, by genetic factors that are yet to be identified. Future genetic and molecular studies will help to a better understanding of the relationship between inflammation and cancerogenesis and may lead to new diagnostic and therapeutic possibilities for those subjects with CP that are at high risk of developing pancreatic carcinoma.

### Conflict of interest statement

The author declares that the research was conducted in the absence of any commercial or financial relationships that could be construed as a potential conflict of interest.
